# Suboptimal Larval Habitats Modulate Oviposition of the Malaria Vector Mosquito *Anopheles coluzzii*

**DOI:** 10.1371/journal.pone.0149800

**Published:** 2016-02-22

**Authors:** Eunho Suh, Dong-Hwan Choe, Ahmed M. Saveer, Laurence J. Zwiebel

**Affiliations:** 1 Department of Biological Sciences, Vanderbilt University, Nashville, Tennessee, United States of America; 2 Department of Entomology, University of California, Riverside, California, United States of America; 3 Department of Pharmacology, Vanderbilt Brain Institute, Program in Developmental Biology, and Institutes of Chemical Biology and Global Health, Vanderbilt University Medical Center, Tennessee, United States of America; New Mexico State University, UNITED STATES

## Abstract

Selection of oviposition sites by gravid females is a critical behavioral step in the reproductive cycle of *Anopheles coluzzii*, which is one of the principal Afrotropical malaria vector mosquitoes. Several studies suggest this decision is mediated by semiochemicals associated with potential oviposition sites. To better understand the chemosensory basis of this behavior and identify compounds that can modulate oviposition, we examined the generally held hypothesis that suboptimal larval habitats give rise to semiochemicals that negatively influence the oviposition preference of gravid females. Dual-choice bioassays indicated that oviposition sites conditioned in this manner do indeed foster significant and concentration dependent aversive effects on the oviposition site selection of gravid females. Headspace analyses derived from aversive habitats consistently noted the presence of dimethyl disulfide (DMDS), dimethyl trisulfide (DMTS) and 6-methyl-5-hepten-2-one (sulcatone) each of which unitarily affected *An*. *coluzzii* oviposition preference. Electrophysiological assays across the antennae, maxillary palp, and labellum of gravid *An*. *coluzzii* revealed differential responses to these semiochemicals. Taken together, these findings validate the hypothesis in question and suggest that suboptimal environments for *An*. *coluzzii* larval development results in the release of DMDS, DMTS and sulcatone that impact the response valence of gravid females.

## Introduction

Mosquito-borne malaria remains among the greatest threats to global human health [1]. Inasmuch as effective vaccines are still elusive, the widespread use of the current set of anti-malarials and insecticides has contributed to the rise in resistance to these agents in both pathogens and vectors, respectively [2, 3]. In this light, vector control remains among the most effective methods in reducing disease transmission [4].

A critical feature of improved vector control programs is an enhanced understanding of the mechanistic basis of both vector competence and vectorial capacity. Together with blood-meal host selection/preference, the search for oviposition sites representing optimal larval breeding habitats are crucial decisions in mosquito reproductive cycles that directly impact vector population size and, accordingly, vectorial capacity [5]. In the course of oviposition site selection in the field, gravid females dynamically process multiple signals including hygroscopic, olfactory, tactile, thermal or visual cues to assess larval breeding sites [6–8].

Gravid *An*. *gambiae* s.l. Giles,which has recently reclassified as *An*. *gambiae* s.s. Giles and *An*. *coluzzii* Coetzee and Wilkerson [9] oviposit directly on a diverse spectrum of habitat water as well as surrounding muds where larvae hatch and find their way to nearby water [10]. Accordingly, immature *An*. *gambiae* s.l. occurs in aquatic breeding sites with diverse biological and physico-chemical characteristics [11–13]. In addition to abiotic factors such as water vapor, hydration, or visual contrast of oviposition sites [14–17], studies have suggested chemical signals derived from biotic components of breeding sites such as microbial larval food sources [18–20] or predators/competitors [21–23] influence the oviposition behavior of gravid *An*. *gambiae* s.l. Indeed, oviposition site selection behavior of mosquitoes has been postulated to evolve toward maximization of offspring fitness interacting with multiple biotic factors [24–28]. Consistent with this hypothesis, oviposition behavior of gravid female mosquitoes is associated with conspecific larval density [27–29], suggesting that population size of immature mosquitoes are maintained at near optimal levels by selective oviposition of gravid females utilizing unknown cues associated with the pre-existing larval population that reflects the expected fitness of those populations. Specifically, presence of conspecifics in low density could be an indication of suitable breeding sites (e.g., appropriate larval food level along with the absence of predators) where oviposition activity tends to be stimulated/increased, whereas increased larval competition for limited resource result in overall reduction in the fitness of progenies where the oviposition activity tends to be deterred/reduced [24, 25, 30, 31].

In order to further characterize aversive oviposition cues specifically associated with suboptimal rearing conditions, we generated artificial habitats containing overcrowded and resource-deprived larvae that would be expected to have a repellent effect on ovipositing females. To examine this, we developed a two-choice oviposition bioassay to evaluate the oviposition preferences of gravid females to pre-conditioned larval water (LW) and characterized its major volatile constituents in both behavioral and electrophysiological paradigms with gravid adult females. Our studies validate the hypothesis that suboptimal larval habitats repel gravid females and identify dimethyl disulfide (DMDS), dimethyl trisulfide (DMTS), and 6-methyl-5-hepten-2-one (sulcatone) as specific semiochemicals involved in modulating the oviposition behavior of our laboratory-colony of *An*. *coluzzii*.

## Materials and Methods

### Mosquito rearing

*Anopheles coluzzii* (SUA 2La/2La) originating from Suakoko, Liberia, previously known as *Anopheles gambiae* s.s. M form [9], was maintained in the Vanderbilt Insectary and mosquito rearing primarily followed a lab protocol developed in a previous study [32]. In brief, larvae were reared under standardized conditions (> 2 cm^2^ water surface per larva with 1 liter of dH2O per larval pan) by adding food *ad libitum* in environmental chamber (27°C, 80% relative humidity, light:dark = 12:12 h) to ensure consistent larval development and large size adults. Pupae were collected and eclosed in a cage, and females and males were allowed to mate with constant access to 10% sucrose solution. For blood feeding, 6 to 7 days old females were provided with human blood (BioChemed, Winchester, VA) by using Hemotek membrane feeding system (Hemotek, Lancaster, UK), and 4.5% CO_2_ was utilized to promote blood feeding.

### Preparation of conditioned larval water (LW)

**Fig 1:**
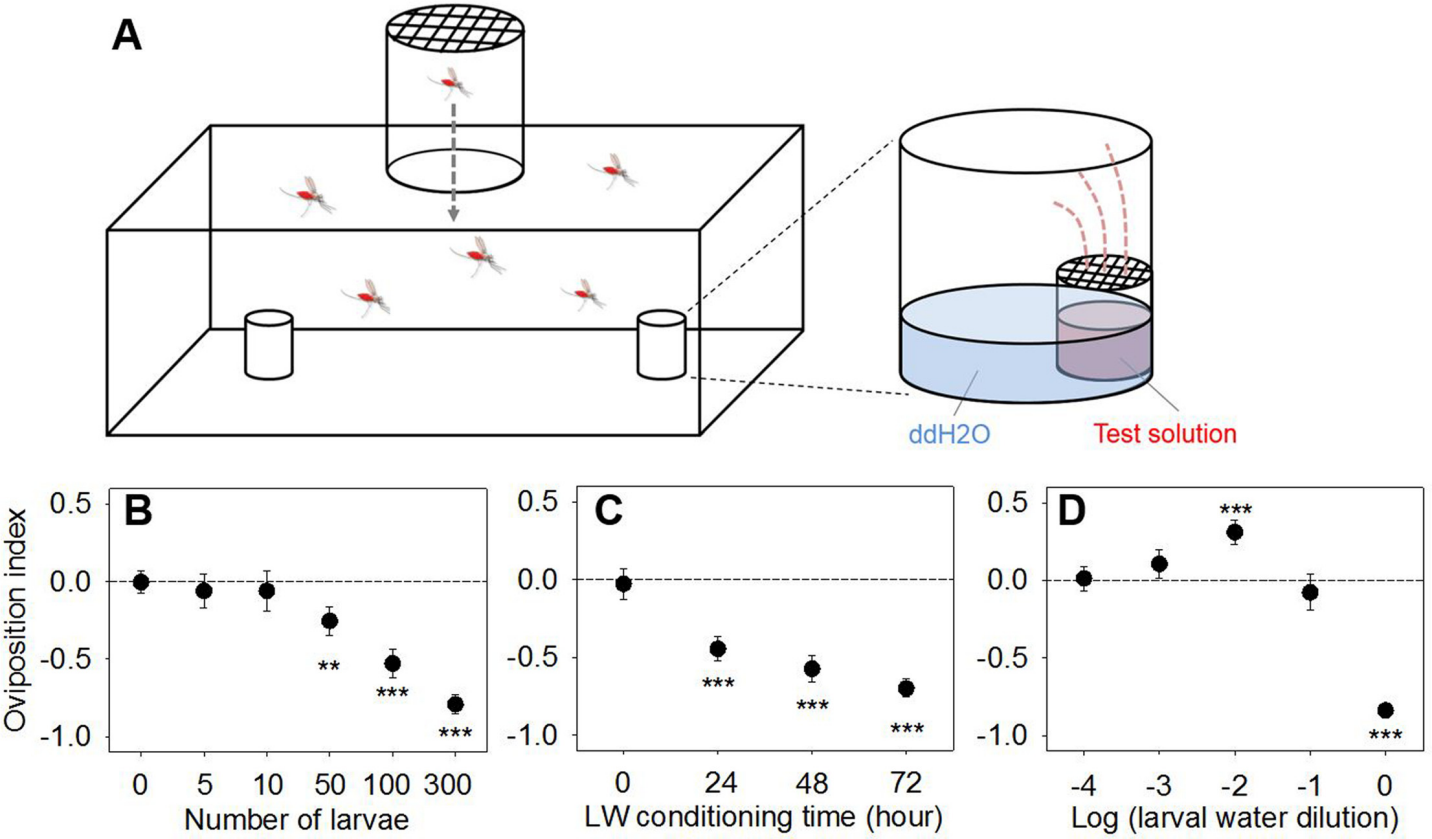
Volatiles from overcrowded/starved larval water habitats affect oviposition behavior of *An*. *coluzzii* gravid females. (A) Schematic of oviposition dual choice behavioral assay designed to examine olfactory-driven responses (see Methods for details). Oviposition preference of gravid females to larval water samples with varied treatment by (B) incubating different number of late instars for 72 h, (C) incubating 300 late instars for different time period, and (D) diluting LW sample obtained by incubating 300 late instars for 72 h. Asterisks represent significant OI values different from zero (***, *p* < 0.001; **, *p* < 0.01; Wilcoxon signed-rank test, two-sided). Error bar = s.e.m. (n = 17 ~ 36).

All LW treatment used larvae reared under standard rearing condition described above. Late 3^rd^ or early 4^th^ instars were collected in dH_2_O (see Fig 1 for preparation of conditioned larval water) and washed/filtered several times through a wire sieve and then transferred into 50 ml borosilicate glass bottle with 20 ml of HPLC grade dH_2_O for incubation at 27°C. Conditions used here for LW treatment were comparable to a previous study [29]. Control water (CW) was prepared using HPLC grade dH_2_O without larvae. Larvae were removed from LW using a metal sieve (standard sieve No. 40; opening size = 0.420 mm) after completion of larval incubation, and larvae were not present in water samples during bioassay periods. Materials used for LW and CW sample preparations (i.e., glass containers) were first cleaned with detergent, and then serially washed with distilled water, methanol and finally methylene chloride. Cleaned materials were incubated at 75°C overnight followed by complete desiccation before use to minimize contamination of random odors. LW and CW samples were kept at 4°C for no more than one week before being used for the oviposition assay and headspace chemical analysis.

### Dual choice oviposition assay

Laboratory based oviposition bioassays were conducted in a growth chamber following the previously established protocol [32]. To prepare gravid female mosquitoes, 6 to 7 days old females were blood fed, and fully engorged females were transferred to a separate cage with constant access to 10% sucrose solution. Preparation of the experimental setup began around 1 hour prior to scotophase in the environmental chamber under the same conditions used for larval rearing as described above. Two days after blood feeding, 10 gravid females were transferred into the “releasing chamber” with a screen on top, and the females were allowed to enter the assay cage (polypropylene, length = 37.8 cm, height = 15.2 cm, width = 13 cm) through a center pathway (diameter = 6 cm) after dark cycle began (see Fig 1A). A borosilicate glass vial (capacity = ~ 1 ml, 14.65 × 19 mm; Qorpak, Bridgeville, PA) with a screen on top (10 × 10 mm) was used to contain 1 ml of test or control water placed in order to exclude the effect of tactile cue on the oviposition behavior of gravid females. The vials were placed inside of the egg cups (PET, top diameter = 4.5 cm, height = 4.1 cm, bottom diameter = 2.9 cm) farther from the releasing chamber with their sides in contact with the inner side of the egg cups. The two egg cups (control and test) were 26 cm apart. The egg cups were filled with 7 ml of ddH_2_O as an oviposition substrate. In this experimental design, gravid females were not allowed to touch aqueous solution within the vial, thus oviposition preference observed in the bioassays was assumed to be driven by olfactory cues. Location of egg cup containing test water was rotated between assay cages, and the assay cages were randomly placed within a larger enclosure (acrylic, 86 × 120 × 86 cm) to minimize external effect on the oviposition preference. For the preparation of test waters of lower concentrations, LW were diluted in ddH2O, and DMDS, DMTS and sulcatone was dissolved and diluted in 0.1% DMSO as standard solutions. DMDS, DMTS, sulcatone and DMSO (≥ 98% purity) were obtained from Sigma-Aldrich, Inc. All assay cages were cleaned by using 70% EtOH and fully dried before each assay. Gravid females were allowed to oviposit during scotophase, and collected eggs were counted in the following morning. All bioassays were conducted using at least three different batches of mosquitoes.The total number of collected eggs were used to calculate oviposition index (OI) using formula OI = (N_t_−N_c_)/(N_t_+N_c_) [33] with N_t_ = number of eggs collected in the egg cup with LW or test volatiles (i.e., larval conditioned water or test compound in the vial), and N_c_ = number of eggs collected in the egg cup with CW volatiles. Oviposition preference of gravid females was determined by OI values using Wilcoxon signed rank test (*p* = 0.05, two-sided; JMP 8.0.1; SAS Institute, Cary, NC), and the nonparametric method was used due to non-normality of data. If the OI values were significantly different from zero with positive or negative values, the subject was considered to have attractant or repellent effect on oviposition behavior of gravid females, respectively. In order to examine any stimulatory or deterrent effect of test water/compounds on the oviposition of gravid females, ANOVA (*p* = 0.05; JMP 8.0.1; SAS Institute, Cary, NC) was used to test the effect of treatment variables (e.g., larval density, incubation time, concentration, etc.) on the total number of eggs collected in a cage after square root transformation to normalize the data as needed. Homogeneity of variance was also confirmed for all data sets. If there’s no effect of treatment variable, we considered oviposition of gravid females were neither stimulated nor deterred by the presence of test water/compounds.

### Head space analysis on pre-conditioned larval water

Headspace volatiles of LW samples (300 early 3^rd^ or late 4^th^ instars incubated for 72 h) were collected with a solid-phase microextraction (SPME) samplers (65 μm polydimethylsiloxane [PDMS] /divinylbenzene [DVB]; Supelco, Inc) by exposing the fiber in the headspace of a glass vial (40 ml) containing 10 ml of the sample for 18 h. Based on preliminary study with multiple fibers (100 μm PDMS, 65 μm PDMS/DVB, 75 μm carboxen/PDMS; Supelco, Inc) with various collection time, the current protocol (i.e., PDMS-DVB for 18 h) for volatile collection was established to maximize the detection sensitivity for the compounds of interest. The volatile collection was conducted at 25–26°C without agitation of the sample. Immediately following the collection, volatiles absorbed in the SPME fiber were analyzed by gas chromatography (GC)–mass spectrometry (MS). Control sample was collected from CW of same amount. For GC-MS, electron impact mass spectra (70 eV) were taken with an Agilent 5975C mass selective detector interfaced to a Agilent 7890A gas chromatograph equipped with a DB-5 column (30 m × 0.32 mm inner diameter, Agilent Technologies). Volatile extracts from SPME fiber were injected in splitless mode, with a temperature program of 50°C for 1 min and then 10°C min^−1^ to 280°C with 5-min hold. The temperature of injector and transfer line was 250°C. Helium was used as the carrier gas. Six different LW and LW headspace samples prepared with different batches of mosquito larvae were analyzed with the same method. Compounds in the samples were identified by comparison of retention times and mass spectra with those of authentic standards. A semi- quantitative estimate of the source concentration for LW volatiles was obtained by comparing the peak integration values from 18h LW headspace collections to those obtained from similar headspace collections from standard solutions (10 ml) containing known concentrations of DMDS, DMTS and sulcatone individually (e.g., 10^−6^ M, 10^−7^ M 10^−8^ M). The ratio of the integration value from the standard preparation that generated the headspace integration value that was most similar to the average integration value of the target compound in LW headspace (i.e., within a range of one order of magnitude) determined the semi-quantitative value of the concentration of that compound in LW. The following equation was used to calculate the source concentration (M) of each target compound in LW.

$$C_{LW}=\frac{I_{St}}{I_{LW}}\times \;C_{St}$$

where *C*_*LW*_ is the estimated source concentration of a target compound in LW, *I*_*St*_ is the headspace integration value of the standard preparation closest to the average integration value of the target compound in LW headspace, *I*_*LW*_ is the integration value of the target compound in LW headspace, *C*_*St*_ is the source concentration of standard preparation that generated the headspace integration value that was closest to the average integration value of the target compound in LW headspace.

### Transcuticular electrophysiology

Electroantennogram (EAG), electropalpogram (EPG), and electrolabellogram (ELG) recordings were made from chemosensory organs of two days post blood fed gravid females (subsequently confirmed to contain mature Christopher stage IV or V embryos). EAG assays were carried out one hour after the initiation of scotophase according to previously described methods [34], and continued for 3 ~ 4 hours with randomized order of EAG, EPG and ELG. Here, a cold anesthetized gravid female was restrained on slide glass using double sided tape with legs and wings removed as previously described [35]. The last segment of antenna was subsequently transected and connected to a recording glass electrode filled with Ringer solution (96 mM NaCl, 2 mM KCl, 1 mM MgCl2, 1 mM CaCl2, 5 mM HEPES, pH = 7.5) where AgCl coated silver wire was in contact to complete a circuit with reference electrode which was similarly connected into compound eye of the female. The antennal preparation was continuously exposed to humidified, charcoal—filtered air flow (1.84 liter/min) transferred through a borosilicate glass tube (inner diameter = 0.8 cm) using stimulus controller (Syntech, Hilversum, The Netherlands), and the open end of the glass tube was located 5 mm from the antennal preparation. Forty microliters of test or control stimuli were transferred onto a piece of filter paper (10 × 50 mm) which was then placed inside of the Pasteur pipette. LW samples (300 early 3^rd^ or late 4^th^ instars incubated for 72 h) were diluted in ddH_2_O, and test chemical was dissolved and diluted in mineral oil to prepare lower concentrations of test stimuli. Odor was delivered to the antennal preparation for 500 ms through a hole placed on the side of the glass tube located 10 cm from the open end of the tube (1.08 liter/min), and the stimulus odor was mixed with continuous air flow through the hole. A charcoal-filtered air flow (0.76 liter/min) was delivered from another valve through a blank pipette into the glass tube at the same distance from the preparation (i.e., 10 cm from the open end of tube) in order to minimize changes in flow rate during odor stimulation. The test sequence of odors (LW, DMDS, DMTS or sulcatone) was randomized and the order of individual stimuli (i.e., different concentrations of odor and control stimulus) was randomized within each odor session with the interval time of > 40 seconds between every stimulus to minimize the effect of changes in odor sensitivity over time. Control odors were stimulated before and after the first session of odor (1-octen-3-ol 10^−1^ M for EAG and EPG, oxovaleric acid 10^−1^ M for ELG) in order to check the response sensitivity of test individuals as these compounds have been described as one of most active compounds for each chemosensory organ in previous studies [35, 36]. The resulting signals were amplified 10× and imported into a PC via an intelligent data acquisition controller (IDAC, Syntech, Hilversum, The Netherlands) interface box, and then recordings were analyzed by EAG software (EAG Version 2.7, Syntech, Hilversum, The Netherlands). Seven recordings were replicated on different individual preparations per odorant. EPG and ELG recordings and analyses were made using a maxillary palp and labellum of gravid female mosquito following the protocol described above for EAG. In order to enhance the response/noise ratio in EPG and ELG, the tip of palp was not modified except removing mechano-sensory sensilla from the last segment of maxillary palp, and a proboscis was restrained on double-sided tape with labrum removed from labellum. For the analysis of response amplitude, control response (ddH2O or oil) was subtracted from the response amplitude of test stimuli. One sample *t*-test was carried out to determine whether the response amplitude (i.e., control response subtracted) was significantly different from zero (*p* = 0.05, one-sided; JMP 8.01, SAS Institute, Cary, NC). One-sided test was used with the assumption that the polarity of odor stimuli (e.g., depolarization or hyperpolarization) is consistent regardless of concentration. All compounds were obtained from Sigma-Aldrich, Inc. with highest purity available.

### Single sensillum electrophysiological recording

Electrophysiological recordings were conducted on single capitate peg (cp) sensillum along the maxillary palp of female mosquitoes that house three types of olfactory receptor neurons (ORNs) [35]. Here, gravid females of *An*. *coluzzi* (subsequently confirmed to contain mature Christopher stage IV or V embryos) were cold immobilized (~1 min at -20°C) and mounted on a double-sided tape on a microscope glass slide (25 × 75 × 1.0 mm). Two glass capillaries inserted with chloridized silver wire of appropriate size and filled with 0.1 M KCl saline were used as reference and recording electrode, respectively. The reference electrode was placed in the eye, and the recording electrode was connected to a preamplifier (10×, Syntech, Hilversum, The Netherlands) and inserted at the base of cp sensillum to record the extracellular activity of the ORNs. The signals were digitized by the IDAC4 interface box (Syntech, Hilversum, The Netherlands) and analyzed with Auto Spike v. 3.2 software (Syntech, Hilversum, The Netherlands).

Odor stimuli were diluted in DMSO in decadic steps, ranging from 10^−5^ M to 1 M and DMSO was used as controls. Odor cartridges were prepared by loading filter paper disk (ca. 12.7 mm ø) with 10 μl of test compounds and inserting them into glass Pasteur pipettes connected via silicone tubing to a stimulus controller (Syntech, Hilversum, The Netherlands). Odor stimulation (0.5 liter/min) was carried out for 500 ms by inserting the tip of the SSR odor cartridge into a glass tube with an a charcoal filtered, humidified air-flow (0.5 liter/min) directed towards the maxillary palp which was positioned 10 mm away from end of the glass tube.

The extracellular activity of an individual cp sensillum that houses three physiologically distinct ORNs, cpA, cpB, and cpC was characterized based on the spike amplitudes, spike frequency, and shape as described in S5B Fig and a previous study [35]. The response of an individual neuron to a stimulus was determined by manually counting number of spikes 1000 ms after the onset of neuronal response minus the number of spikes 1000 ms prior to stimulus onset. To rule out the solvent (DMSO) response, solvent spikes were subtracted from the odor induced spike counts. One sample *t*-test (*p* = 0.05, two-sided; JMP 8.01, SAS Institute, Cary, NC) was used to determine whether the normalized response value (i.e., solvent response subtracted) for each neuron was significantly different from zero for each concentration of odor stimuli. If the response value was significantly greater or smaller than zero, the neuronal response was considered as excitatory or inhibitory.

## Results

### Oviposition behavior of *An*. *coluzzii* is mediated by larval water-derived volatiles

A series of oviposition assays were conducted to examine the effects of high larval density coupled with limited nutrient resources on the oviposition behavior of *An*. *coluzzii* gravid females. Here, studies utilized a range of LW treatments that varied the duration of time without food and larval density to establish conditions that would elicit the strongest effects on olfactory-driven oviposition behaviors of gravid females.

In the first experiment, preconditioned LW samples were obtained by varying the number of late instars (5, 10, 50, 100, 300 larvae) incubated for 72 hours and were used in dual choice oviposition bioassays between egg laying cups containing either LW (treatment) or control water (CW, untreated). Aversive responses (as indicated by negative oviposition index [OI] values) that reduced oviposition increased relative to the number of larvae in LW treatments with OI values ranging from -0.25 to -0.79 (50 to 300 larvae, Fig 1B). Lower larval densities (5 and 10 larvae) resulted in LW samples with no effect on the oviposition preference of gravid females (Fig 1B). Importantly, the number of total eggs collected from two oviposition cups in the dual choice bioassay was not affected by the initial number of larvae used for LW conditioning (ANOVA, *F*_*5*,*164*_ = 1.61, *p* = 0.16; S2A Fig) suggesting the presence of compounds in the assay cages neither stimulated nor deterred oviposition of gravid females. In these studies, the surviving larvae were counted to estimate larval survival for each LW treatment which was significantly reduced (54%) at higher larval densities (300 larvae) while lower densities (5, 10, 50, 100 larvae) displayed similar larval survival levels ranging from 94% to 100% (S1A Fig). We next varied incubation time (0 h, 24 h, 48 h, 72 h) of the highest larval density (300 larvae) to generate LW samples. Significant aversion was observed in response to LW generated when larvae were held beyond 24 h with oviposition index (OI) values ranging from -0.45 to -0.7 while no effect was observed for CW treatments (Fig 1C), indicating the repellent effect of LW on gravid females is positively correlated with both initial larval density and the duration of LW conditioning. Once again, the overall number of total eggs collected from the two oviposition cups in the dual choice assay was not affected by duration of the LW conditioning (ANOVA, *F*_*3*,*123*_ = 0.96, *p* = 0.42; S2B Fig). In these studies, larval survival was significantly reduced over time ranging from 94% to 52% and increasing numbers of dead larvae (often with lost body parts) were observed and removed in the course of daily visual inspections (S1B and S1C Fig). In addition, LW samples with increased larval densities, varied conditioning time or larval age showed no additional increase in OI value (S3 Fig) as compared to maximum OIs observed in the previous bioassays. Thus, together with the results that the LW samples generated from 300 larvae incubated for 72 hours exhibited significantly increased larval mortality (S1A and S1B Fig), LW samples generated in this manner were used as standard LW for the duration of this study.

This concentration dependent effect was also examined by observing the behavioral responses elicited by serial dilutions (in ddH_2_O) of standard LW samples in dual choice oviposition bioassays. Consistent with prior results, significant aversion was observed in oviposition bioassays using undiluted LW (OI = -0.84 ± 0.049; mean ± s.e.m; n = 29; Wilcoxon signed-rank, *Z* = -216, *p* < 0.0001) although this effect was not observed when LW was diluted 10 fold (Fig 1D). Interestingly, when LW samples were further diluted to 100 fold, a significant degree of attraction was observed (OI = 0.31 ± 0.079; n = 33; Wilcoxon signed-rank, *Z* = 189; *p* < 0.001), and once again, this effect decreased as the concentration of LW was further reduced (Fig 1D). As in previous assays, the total number of eggs collected from the two oviposition cups was not affected by the concentration of conditioned LW (ANOVA *F*_*4*,*154*_ = 0.76, *p* = 0.55; S2C Fig), suggesting a shift in the distribution of the available eggs.

### Oviposition behavior of *An*. *coluzzii* is affected by larval water specific single compounds

**Fig 2:**
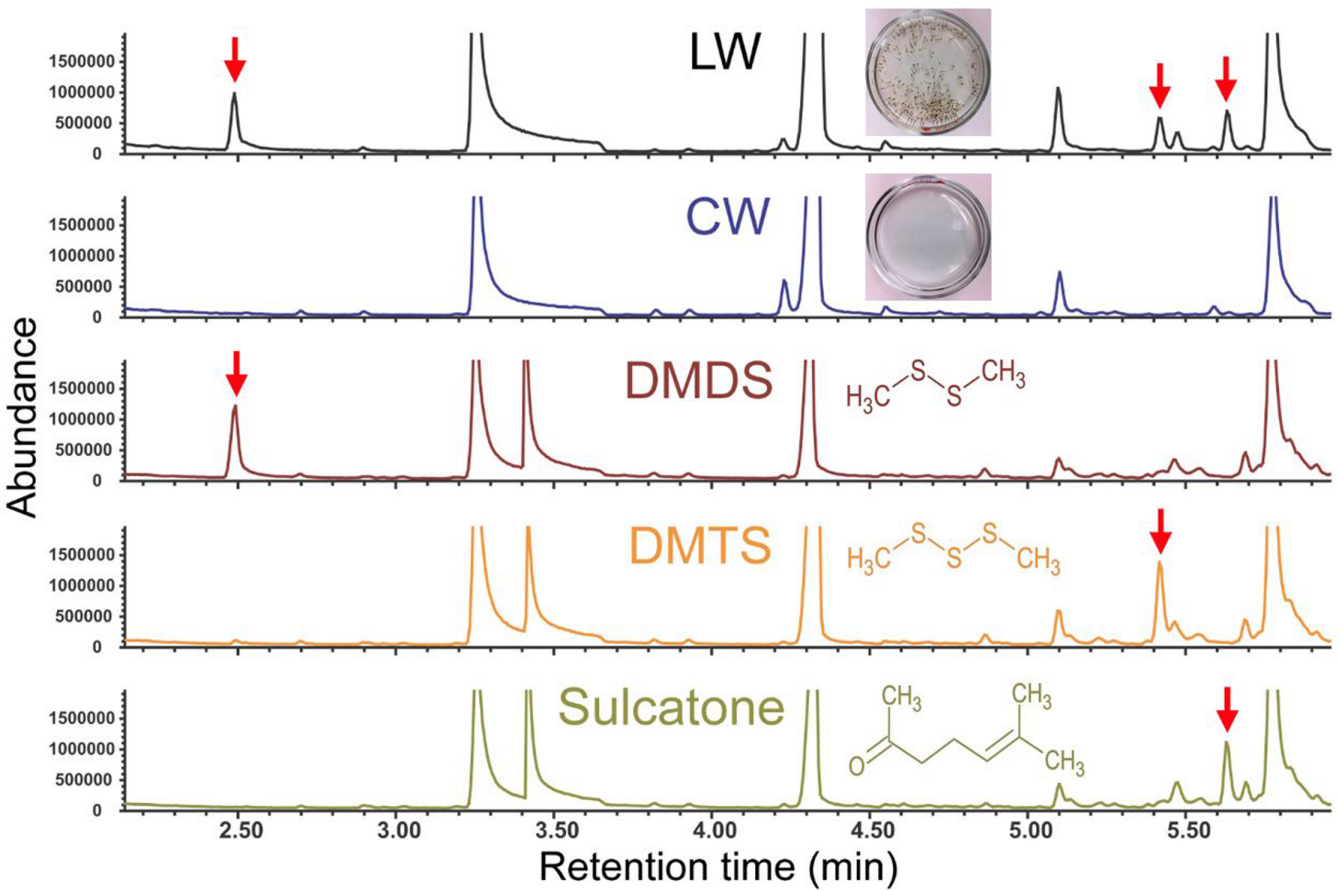
DMDS, DMTS and sulcatone are significant volatile components in the headspace of larval water samples. Partial chromatograms are shown for volatile samples taken from larval water (LW), control water (CW), and standard DMDS (10^-7^ M), DMTS (10^−8^ M) and sulcatone (10^−8^ M) using SPME headspace analysis coupled with gas chromatography–mass spectrometry. Peaks for DMDS, DMTS and sulcatone are marked with red arrows. No additional LW specific compounds were detected beyond the retention time of sulcatone. Large peaks with retention times of approximately 3.2, 4.3 and 5.8 min represent impurities possibly introduced during sample preparations and/or chemical analyses, which are present in all samples including LW and CW.

Headspace analyses of LW samples were carried out using gas chromatography (GC)—mass spectrometry (MS) with solid phase micro-extraction (SPME) fibers. In addition to several trace compounds, significant concentrations of dimethyl disulfide (DMDS), dimethyl trisulfide (DMTS) and 6-methyl-5-hepten-2-one (sulcatone) were consistently detected in the headspace of LW samples, but not in the headspace of CW samples (Fig 2). A semi-quantitative analysis of the headspace of standard solutions containing known amounts of individual compound indicated that 10^−7^ M DMDS, 10^−8^ M DMTS and 10^−8^ M sulcatone in aqueous preparations produced the peak integration values that were most similar to those observed in LW headspace (i.e., within a range of one order of magnitude) (Fig 2). Based on the ratio between peak integration values from headspaces derived from compound standards at those concentrations and those integration values derived from the corresponding peaks from six LW headspace samples (see Methods for detailed information), the approximate concentrations of DMDS, DMTS and sulcatone in LW were estimated to be (1.72 ± 0.47) × 10^−7^, (3.55 ± 0.49) × 10^−9^, and (2.75 ± 1.07) × 10^−9^ M (mean ± s.e.m; n = 6), respectively.

**Fig 3:**
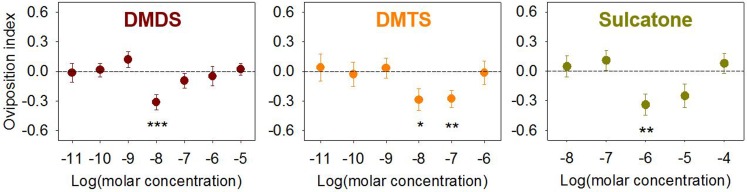
Oviposition behavior of *An*. *coluzzii* gravid females is negatively affected by DMDS, DMTS and sulcatone. *An*. *coluzzii* gravid females were allowed oviposit between control water and DMDS, DMTS and sulcatone with serial dilutions. Asterisks represent significant OI value different from zero (**, *p* < 0.01; *, *p* < 0.05; Wilcoxon signed-rank test, two-sided). Error bar = s.e.m. (n = 14 ~ 35).

The behavioral responses of gravid female *An*. *coluzzii* to egg laying cups containing DMDS, DMTS and sulcatone over various concentrations was examined in dual choice oviposition bioassays. In these studies, egg laying cups with 10^−8^ M elicited a significant aversive response with OI value of -0.31 ± 0.078 (mean ± s.e.m.; n = 36; Wilcoxon signed-rank, *Z* = -218, *p* < 0.001), while 10^−9^ M DMDS elicited potentially attractant effects with *p* value approaching statistical significance (OI = 0.12 ± 0.081; n = 39; Wilcoxon signed-rank, *Z* = 120, *p* = 0.093) (Fig 3). DMTS and sulcatone elicited aversive responses in a similar dose dependent manner. Here, 10^−8^ M and 10^−7^ M DMTS had significant repellency with OI values of -0.29 ± 0.11 (n = 27; Wilcoxon signed-rank, *Z* = -73, *p* < 0.05) and -0.28 ± 0.087 (n = 18; Wilcoxon signed-rank, *Z* = -59, *p* < 0.01), respectively. Similarly, sulcatone elicited significant aversive responses at 10^−6^ M (OI = -0.34 ± 0.11; mean ± s.e.m.; n = 21; Wilcoxon signed-rank, *Z* = -77, *p* < 0.01) while the OI value at 10^−5^ M sulcatone approached significance (-0.24 ± 0.12; n = 22; Wilcoxon signed-rank, *Z* = -53, *p* = 0.085) (Fig 3). The total number of eggs collected from both oviposition cups was not affected by different concentrations of DMDS, DMTS and sulcatone (ANOVA, *p* > 0.05; S2 Fig).

### The antennae, maxillary palp and labellum of gravid mosquitoes respond to larval water, dimethyl disulfide, dimethyl trisulfide and sulcatone

**Fig 4:**
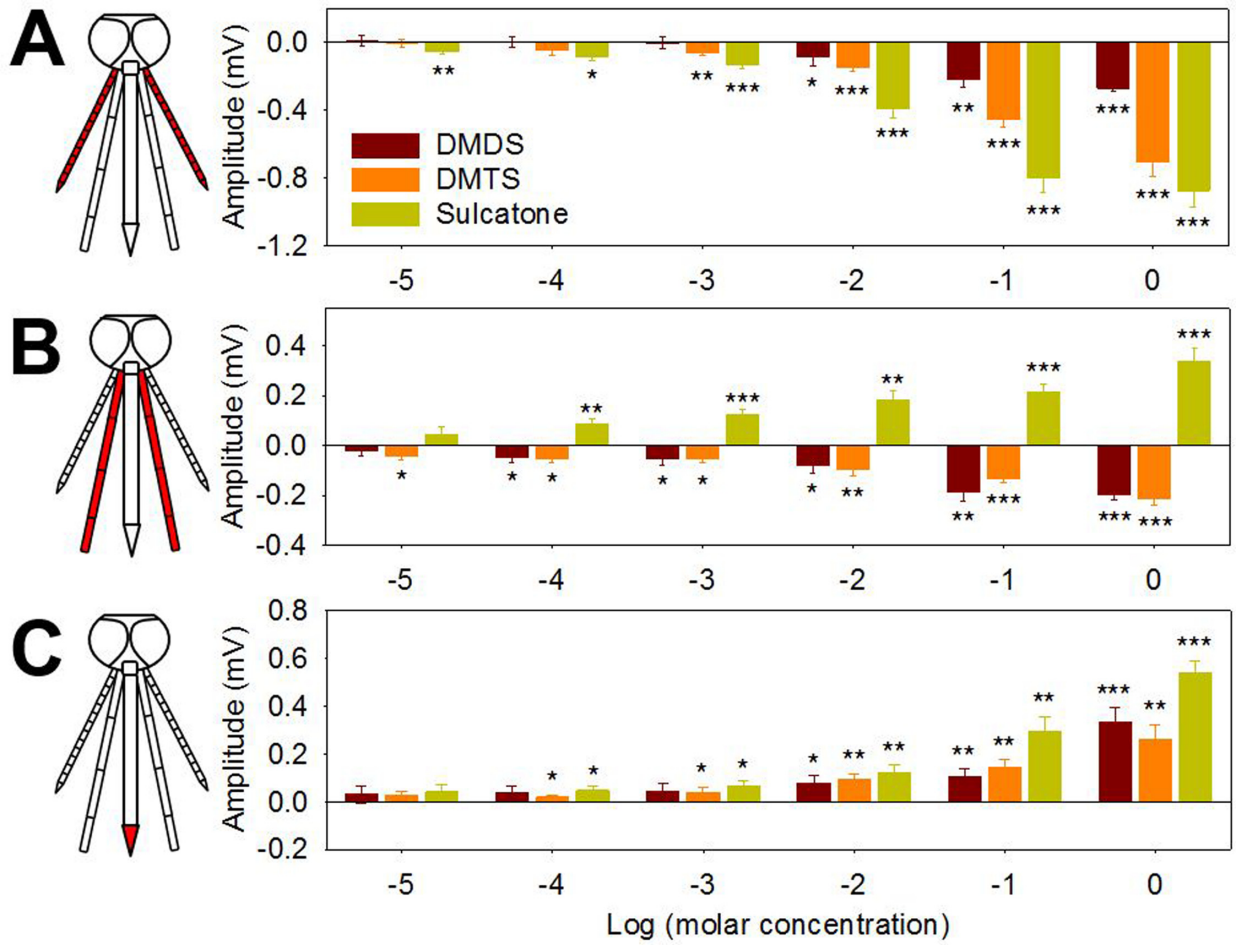
Chemosensory appendages of gravid *An*. *coluzzii* electrophysiologically respond to DMDS, DMTS and sulcatone. (A) EAG, (B) EPG and (C) ELG responses (each chemosensory organ is highlighted in red in a schematic diagram of mosquito head) are expressed as response difference to solvent control (oil) of *An*. *coluzzii* females to DMDS, DMTS and sulcatone. Y axis represents response amplitude subtracted by control values and X axis represents log transformed molar concentration. Asterisks represent significant response amplitude different from zero (***, *p* < 0.001; **, *p* < 0.01; *, *p* < 0.05; one sample *t*-test, one-sided). Error bar = s.e.m. (n = 7).

In an effort to further explore the basis of DMDS, DMTS and sulcatone on *An*. *coluzzii* oviposition preferences we examined the electrophysiological responses from the antennae, maxillary palp and labellum of gravid mosquitoes. These responses were initially characterized using electroantennogram (EAG), electropalpogram (EPG) and electrolabellogram (ELG) assays that measure transcuticular voltage changes derived from collective neuronal responses [37]. In these studies, each chemosensory appendage exhibited significant, albeit differential, sensitivity to individual stimuli (Fig 4) as well as to complex LW samples (S4 Fig). Specifically, the maxillary palp maintained a significant response to a 10 fold LW dilution while antennal and labellar responses were restricted to only undiluted LW (S4 Fig). Similarly, the antennae of gravid *An*. *coluzzii* was considerably more sensitive to sulcatone with significant responses at 10^−5^ M as compared to DMDS or DMTS where the response thresholds were 10^−2^ M and 10^−3^ M, respectively (Fig 4A). The maxillary palp was somewhat more sensitive to DMDS and DMTS with significant responses as low as 10^−4^ M and 10^−5^ M, respectively, while significant sulcatone responses were observed at as low as 10^−4^ M (Fig 4B). The labellum was more sensitive to sulcatone and DMTS relative to DMDS with significant responses as low as 10^−4^ M, respectively (Fig 4C). Beyond those threshold levels, the chemosensory appendages exhibited odor-dependent polarization showing diverse response traces (S5A Fig). While all three compounds elicited downward (depolarization) responses in EAGs studies, upward (hyperpolarization) responses were frequently observed in EPG and/or ELG studies (Fig 4 and S5A Fig).

### Neurons in capitate peg sensillum of the maxillary palp respond to dimethyl disulfide, dimethyl trisulfide and sulcatone

**Fig 5:**
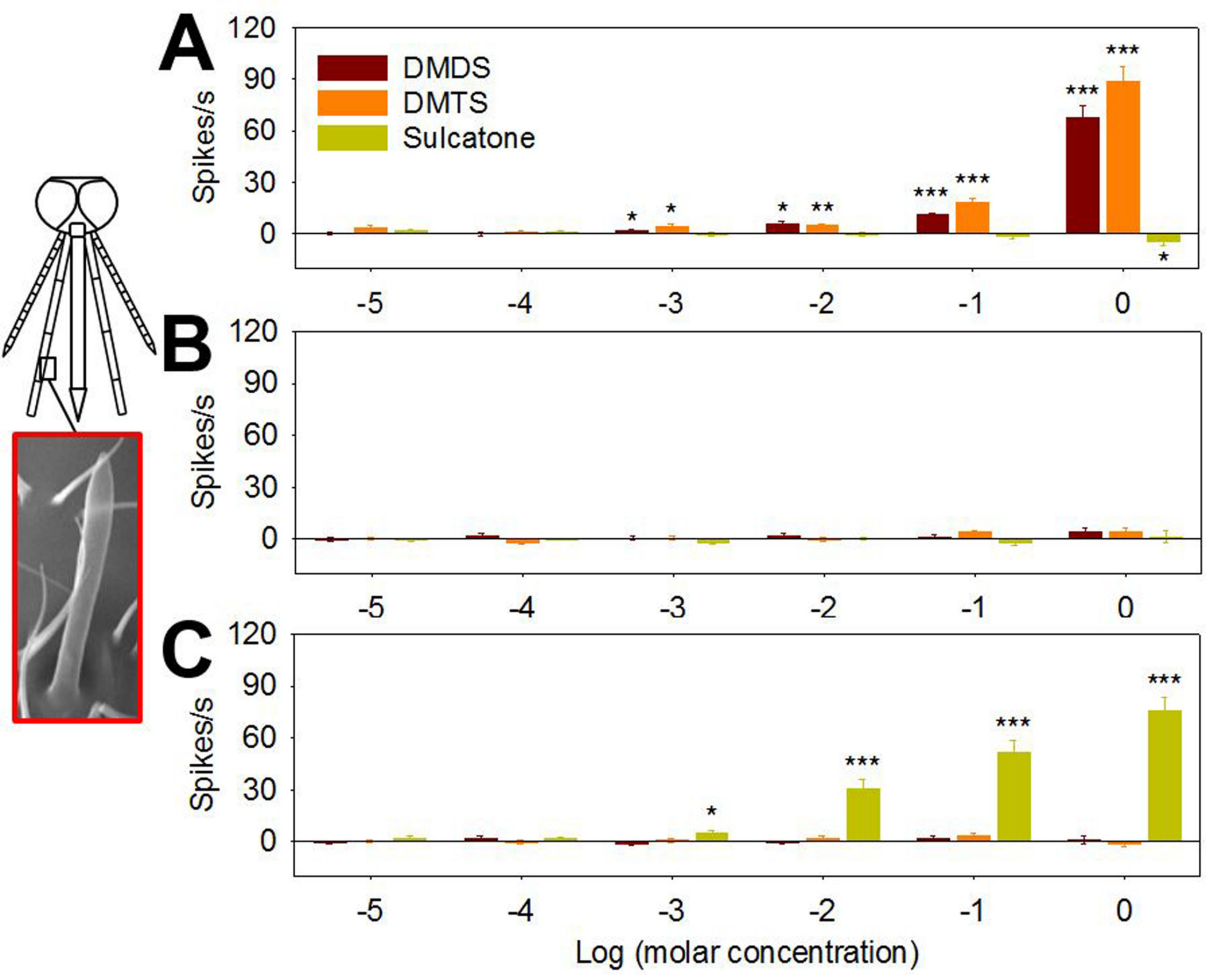
Neurons in capitate peg sensillum of maxillary palp are activated by DMDS, DMTS and sulcatone. Electrophysiological activities of (A) cpA, (B) cpB and (C) cpC neurons in capitate peg sensillum (highlighted in a red box; picture modified from a previous study [35]) of maxillary palp in gravid *An*. *coluzzii* females are identified by spike amplitudes, and changes in spike frequency are quantified to characterize individual neuronal response to varying concentrations of DMDS, DMTS and sulcatone. Y axis represents response spike number subtracted by control response values (DMSO) and X axis represents log transformed molar concentration of DMDS, DMTS and sulcatone. Asterisks represent significant response amplitude different from zero (***, *p* < 0.001; **, *p* < 0.01; *, *p* < 0.05; one sample *t*-test, one-sided). Error bar = s.e.m. (n = 7~10).

We next utilized single sensillum electrophysiological recordings (SSR) to provide a detailed characterization of the neuronal responses to DMDS, DMTS and sulcatone on the maxillary palp of gravid *An*. *coluzzii*. Here, well characterized capitate peg (cp) sensilla containing triad chemosensory neuron subpopulations (cpA, cpB and cpC) are uniformly distributed [35]. Spike sorting analyses revealed dose-dependent responses for cpA neurons which showed significantly increased spike frequencies in response to as low as 10^−3^ M DMDS and DMTS (Fig 5A). In contrast, cpB (Fig 5B) and cpC (Fig 5C) ORNs did not respond to DMDS or DMTS. Unlike DMDS or DMTS, sulcatone elicited significantly increased spike frequencies for cpC neurons in a dose dependent manner down to 10^−3^ M (Fig 5C) while cpA (Fig 5A) and cpB (Fig 5B) neurons displayed no response to sulcatone apart from the significant inhibition of the cpA neuron by 1 M sulcatone (Fig 5A). In these studies, we consistently observed biphasic responses such that stimulation with DMDS and DMTS initially evoked excitatory increases in the CO_2_ sensing cpA neuron [35] spike frequency (S5B Fig). This was followed by discrete inhibitory responses of 0.2 ~ 0.3 sec in which cpA spike activity was reduced before the recovery of spontaneous (base line) neuronal activity (S5B Fig). Unlike DMDS or DMTS, sulcatone elicited a prolonged cpC odorant receptor neuron (ORN) excitatory response for several seconds after the onset of stimulus while cpA ORN responses were inhibited for more than one second (S5B Fig).

## Discussion

Despite many studies that posit the role of chemical signals in mediating oviposition site selection of *An*. *gambiae* s.l., a paucity of validated (in either laboratory or field studies) oviposition-related semiochemicals have been characterized in this medically important mosquito. Indeed, at this point and apart from the general role of water vapor as an attractant [16] only three volatile, single-molecule oviposition cues have been identified in *An*. *gambiae* s.l.: 2-propylphenol and 4-methylcyclohexanol for *An*. *coluzzii* [32], and cedrol for *An*. *gambiae* s.l. [38]. We now report studies that utilize a laboratory-based oviposition bioassay specifically designed to examine olfactory driven behavioral responses of ovipositing females to test the generally held hypothesis that unsuitable conditions for larval development results in the release of semiochemicals that repel or otherwise create aversive signals for gravid female mosquitoes in search of oviposition sites. Taken together, our data support the hypothesis and show that potential oviposition sites conditioned by larvae maintained under poor conditions are actively avoided by gravid female mosquitoes. Consistent with prior studies [29, 39], the degree of repellency was dependent on conditioning characteristics such as duration and larval density (Fig 1). These signals appears to arise strictly as a result of overcrowding as increases in larval density (e.g. from 10 to 50/dish) significantly induced an aversive effect (Fig 1B) without affecting larval survival (S1A Fig). Furthermore, LW-derived effects increased along with incubation time (Fig 1C) coinciding with a decrease in the larval survival rate from 94% to 52% (S1B Fig). This suggests that increased incubation time and density of deceased larval remains may additively increase to the aversive effects of LW on oviposition. While density dependent larval survival and development is well studied in mosquitoes, increased mortality could be also due to larval cannibalism which is reported in *An*. *coluzzii* as also being directly correlated with larval density [40, 41]. Therefore while oviposition in heavily pre-occupied larval habitats may indeed also increase the risk of conspecific larval competition as well as cannibalism, our study does not distinguish between those scenarios. Instead our data suggests oviposition sites are not rejected by gravid females when larval densities are low enough such that the presence of thriving larvae and the absence of aversive signals may be perceived by gravid females as suitable larval habitats. Identification of the precise source(s) of volatile compounds should provide useful information whether the aversive nature of the standard LW used in this study is due to the release of volatiles as a result of larval starvation/crowding effects or through increase in the concentration of volatiles generated by other constituents in LW (e.g., micro-organisms, etc.).

While the inability of conditioned LW samples produced at lower larval densities to attract gravid females (Fig 1B) is inconsistent with the “dual effect” mosquito oviposition regulation model that balances both attraction and repulsion [28], similar dual effects were observed using serial dilutions of LW samples that elicited the strongest aversive effects (Fig 1D). This suggests that increased larval density may generate not only quantitatively but also qualitatively different odor profiles compared to those derived from LW generated at lower larval densities. This suggests that gravid female *An*. *coluzzii* in the field might be attracted to larval habitats that were previously occupied by a large number of conspecific larvae where volatile cues including DMDS accumulate as a result of larval starvation/crowding effects that have been subsequently diluted with fresh water (as a result of irrigation and/or rain fall) while virgin or under-populated oviposition sites remain relatively neutral. This hypothesis is also supported by a previous oviposition study in which a preference was frequently observed for LW samples generated by using field collected water from natural larval habitats while neutral effects or modest repellency was observed from LW samples using only distilled water [29].

While we cannot, as yet, define the precise source of these chemical signals it is evident that our laboratory-based LW conditioning paradigm induces high larval stress and mortality that likely releases and/or promotes the accumulation of a range of oviposition semiochemicals. Indeed, the process of LW conditioning is likely to liberate a diverse microbiological population derived from damaged, decayed or predated larvae that also constitutes a potential source of semiochemicals. In that light, it is noteworthy that gravid *An*. *coluzzii* was significantly attracted by volatiles emitted by several bacteria species derived from larval midgut or natural larval habitats [19]. In particular, *Stenotrophomonas maltophilia* isolated from natural larval habitats has been identified to repel gravid *An*. *coluzzii* [20]. Similarly, laboratory-based oviposition of *Ae*. *aegypti* is also affected by bacterial volatiles in dose dependent manners [42, 43]. Overall, in addition to diverse effects of a large number of non-olfactory factors (e.g., tactile, visual cues, etc.) [14, 15, 17, 44], oviposition in *An*. *coluzzii* is likely to also be associated with the complex chemical ecology of native oviposition sites that, in part, reflects the population dynamics of microorganisms and pre-adult stages of the mosquito.

We have identified DMDS, DMTS and sulcatone as significant and specific volatile components of our laboratory-derived LW semiochemical blend that has dose-dependent oviposition effects on gravid female *An*. *coluzzii*. These compounds are well-established semiochemicals for a number of insect taxa including host seeking hematophagous insects [45–50]. In particular, DMDS and DMTS have been reported as volatile components in the head-space of bacterial isolates associated with natural larval habitats of *An*. *coluzzii* [19], suggesting a potential role of these compounds in the oviposition site selection under natural conditions. Sulcatone is often considered as a repellent or “masking” odorant in host-seeking mosquitoes [51–53], and the oviposition data presented here is consistent with that mode of action. However, it should be stressed that at this point, we cannot conclude these compounds either as individual compounds or within blends, are necessary and sufficient for the complete spectrum of aversive effects of LW.

Estimated LW source concentrations of DMDS, DMTS and sulcatone ranged from 10^−9^ M to 10^−7^ M, which was overlapping or close to the bioactive concentrations that showed aversive effects in the behavioral assays of unitary compounds (10^−8^ M to 10^−6^ M; Fig 3). While additional studies are required to fully understand the contribution of each of these compounds to the aversive effects of LW (e.g., source concentrations, temporal emission dynamics etc.), the discrepancy between our estimated LW source concentration and the actual bioactive concentrations also reflect the inherent differences between chemical analyses and behavioral bioassays. During the chemical analyses, the extraction devices (i.e., SPME fibers in sealed sample vials) were exposed in a static headspace within the vial in which the volatiles were equilibrated between the gas phase and the liquid phase, producing a partial pressure. In contrast, during the behavioral bioassays, the test organisms (i.e., gravid female in the oviposition arena) were exposed to volatiles that were not contained in a sealed vial, most likely experiencing a freely diffusing concentration gradient within an odor plume. These considerations may partially explain the discrepancy between the estimated LW source concentration of sulcatone (~10^−9^ M) and its oviposition bioassay active concentration (~10^−6^ M) (Fig 3). Furthermore, inasmuch as oviposition could occur anytime during the overnight bioassay [54], this behavior is likely to be affected by distinct headspace volatiles as they are continuously being emitted from the egg cups; an effect which would be expected to contribute to the narrow responsive range of bioactive concentrations resulting in sudden shifts in oviposition preference (Fig 3) as often observed in prior studies [42, 55, 56]. The estimated concentrations must, therefore, represent only the approximate abundance of these compounds in LW. The absence of significant behavioral effects of tertiary mixtures of DMDS, DMTS and sulcatone at the estimated concentrations (data not shown) is not surprising considering the potential limitations in concentration estimations as well as the inherent difficulty to precisely mimic complex nature of LW where vaporization dynamics of individual odors during overnight oviposition assay periods are unknown.

We observed significant dose and appendage-dependent transcuticular electrophysiological responses from the antenna, maxillary palp, and labellum of gravid *An*. *coluzzii* to complex volatiles emitted from undiluted pre-conditioned LW as well as individually to DMDS, DMTS, sulcatone (Fig 4). It is noteworthy that EAG responses to natural larval habitats have previously been reported in *An*. *coluzzii* suggesting that volatiles from those habitats may affect oviposition behavior of gravid females [57]. This suggests that modulation of oviposition behaviors in *An*. *coluzzii* is associated with multiple chemosensory structures each differentially responding to each compound although, at this point, the relative contribution of each chemosensory appendage or cell type in mediating *An*. *coluzzii* oviposition behaviors has yet to be precisely mapped.

While typical EAG responses, represented as downward (depolarization) traces and negative amplitudes were indeed observed for LW and DMDS, DMTS and sulcatone, upward (hyperpolarization)/positive amplitude responses were also observed in EPG and/or ELG in a dose-dependent manner (Fig 4 and S5A Fig). While the precise mechanistic basis underlying this effect remains unresolved, it is noteworthy that similar hyperpolarization responses have also been observed in *An*. *coluzzii* where isovaleric acid, butylic acid, oxovaleric acid, and acetic acid elicited upward ELG responses [36]. Furthermore, several acidic compounds also elicited reversed, hyperpolarized responses in EAG and EPG in a coleopteran larvae, *Melolontha melolontha* [58] suggesting response polarity may be odorant/chemosensory organ specific.

Interestingly, significant behavioral responses of gravid females to the three compounds were observed at relatively lower concentrations (e.g., 10^−8^ to 10^−6^ M) in dual choice oviposition assays (Fig 3) while electrophysiological responses were limited to higher concentrations (e.g., DMDS or DMTS) (Figs 4 and 5). These results suggest oviposition behaviors could be elicited by continuous exposure of volatiles at concentrations that are not necessarily sufficient to evoke EAG/EPG/ELG responses. Electrophysiology studies which measure response profiles of individual appendages or sensory neurons are presumed to display a significantly reduced response sensitivity relative to the intact insect where evolution has generated a highly efficient biological platform (i.e. the central nervous system and other networks) to amplify, integrate and otherwise process numerous signals from peripheral sensory systems to generate highly sensitive behavioral outputs [59]. For these reasons, as well as a range of technical considerations, some of which were discussed above, the responsive thresholds observed in electrophysiological studies are not necessarily expected to reflect the behaviorally active semiochemical concentrations established in organismal bioassays.

While a comprehensive characterization of antennae and labellum SSR responses is challenging for *Anopheles* due to the complexity in sensillar type and location, the maxillary palp provides an ideal model for such studies. This sensory appendage expresses a considerably more narrow cellular and molecular receptor repertoire across a uniform population of capitate peg sensillum containing only three (cpA, cpB and cpC) sensory neurons [35]. DMDS and DMTS induced excitatory responses as well as post-stimulation inhibition in the CO_2_-sensitive cpA neuron of gravid *An*. *coluzzii* (Fig 5A and S5B Fig) [35]. These cpA responses were significantly more sensitive at lower concentrations of DMDS and DMTS (Fig 5A) while both the cpB or cpC neurons were not responsive (Fig 5B and 5C). These data implicate the cpA neuron as a major sensing neuron for DMDS and DMTS on the maxillary palp of *An*. *coluzzii*. While devoid of *An*. *coluzzii* odorant receptor (*Or)* expression, the cpA neuron is known to express three gustatory receptors *Gr22*, *23*, *24* that are principally involved in responses to CO_2_ and have been shown to display sensitivity to a range of other semiochemicals [35, 60]. In contrast, sulcatone induced dose dependent, excitatory responses in cpC neurons (Fig 5C and S5B Fig), which in *An*. *coluzzii* specifically expresses *Or*28 linking that receptor as a putative sulcatone-responsive *Or* on the maxillary palp of *An*. *coluzzii* females. Not surprisingly, functional characterization of *Or28* in Xenopus oocytes has indeed revealed dose-dependent sulcatone responses [35]. The cpC and cpB responses are discriminated based on amplitude differences and the shape of spikes (S5B Fig). Interestingly, dose dependent cpC tonic responses of up to 10 seconds (S5B Fig) were observed while cpA neurons were significantly inhibited for ~one second after high-concentration stimulation with 1 M sulcatone (S5B Fig). The inhibition of cpA neurons with the simultaneous excitatory response of cpC neurons is possibly due to ‘ephaptic coupling’ between these two neurons in which neuronal activities are synchronized by non-synaptic communications as seen in *Drosophila* ORNs [61].

At the molecular level, there are a large number of sulcatone-tuned *Ors* expressed across all three chemosensory appendages that together are likely to be associated with the sulcatone responses of gravid *An*. *coluzzii* females observed here [62, 63]. While DMTS receptors have thus far not been molecularly identified, the distinct cpA-centered neuronal response among maxillary palp neurons elicited by this compound and DMDS suggest they are encoded by either the gustatory or ionotropic receptors expressed in those non-*AgOr* expressing cells on the maxillary palp of *An*. *coluzzii* [35, 60, 64]. Elucidation of the precise relationships between the molecular receptors and these oviposition behaviors is likely to be an important component in the development of vector control strategies that target this critical step in the reproductive lifecycle of *An*. *coluzzii*.

This study provides evidence that olfactory driven oviposition behaviors are modulated by volatiles associated with suboptimal larval breeding sites under laboratory conditions. Specifically, starvation and/or over-crowding of larvae increased the emission of volatile semiochemicals that elicited aversive effects on ovipositing gravid females whereas diluted LW also elicited attractant effects in keeping with a widely accepted mosquito oviposition regulation model [28]. We have identified DMDS, DMTS and sulcatone as distinct, behaviorally active components of this response that elicit dose-dependent behavioral effects, and propose these compounds are likely to be associated with regulating oviposition behavior of *An*. *coluzzii*. Questions as to how the olfactory components of diverse populations of *An*. *coluzzii* revealed in this study fit into the complex dynamics of oviposition biology within natural populations of *An*. *coluzzii* or *An*. *gambiae* s.l. under diverse field conditions remain unanswered and ideally should be addressed in future studies.

## Supporting Information

S1 Fig Survival of *An*. *coluzzii* larvae in overcrowded/starved larval habitats. (A) Different number of larvae were starved for 72h and (B) 300 larvae were starved for differing time period. Differing letters indicate statistical difference at *p* = 0.05 (ANOVA, Tukey post-hoc HSD test). (C) Visual observation of 300 larvae held in 20 ml HPLC water without larval food at four different time points. Red arrows indicate examples of dead larvae at 72h time point.
(TIF)

S2 Fig Total number of eggs collected from dual choice oviposition assays. Number of total eggs did not differ by treatment variables (e.g., larval water treatments, concentration of test compounds, etc.). Error bar = s.e.m. Refer number of replicates from Fig 1 and Fig 3.
(TIF)

S3 Fig Behavioral response of gravid females of *An*. *coluzzii* in oviposition dual choice assay. Additional bioassays using varied treatments in number of larvae, age of larvae, and conditioning time showed similar degree of repellent effects as shown in Fig 1. Error bar = s.e.m. (n = 5 ~ 6).
(TIF)

S4 Fig Electrophysiological responses of chemosensory appendages of gravid *An*. *coluzzii* to volatiles from larval water. Responses are expressed as response difference to water control (ddH_2_O) of *An*. *coluzzii* females to larval water (300 larvae incubated for 72 h). Y axis represents response amplitude subtracted by control values and X axis represents log transformed larval water dilution. Asterisks represent significant response amplitude different from zero (***, *p* < 0.001; **, *p* < 0.01; *, *p* < 0.05; one sample *t*-test, one-sided). Error bar = s.e.m. (n = 7).
(TIF)

S5 Fig Representative traces in electrophysiology studies. (A) Differential response kinetics for each odorant (10^−1^ M or undiluted standard larval water)–chemosensory organ combination in EAG/EPG/ELG (top to bottom; each chemosensory organ is highlighted in red in a schematic diagram of mosquito head) and arrows indicate upward responses. (B) Single-sensillum recordings of the responses of the maxillary palp capitate peg sensilla (highlighted in a red box; picture modified from [35]) of gravid *An*. *coluzzii* females to DMSO, DMDS, DMTS and sulcatone. Action potentials from different neurons are labelled A, B, or C according to spike amplitude and shape. Dark horizontal and vertical bars represent the 500 ms stimulus and 2 mV amplitude, respectively.
(TIF)
