# Supplementary figures and images for: Suboptimal Larval Habitats Modulate Oviposition of the Malaria Vector Mosquito Anopheles coluzzii
Source: PLoS One. 2016 Feb 22;11(2):e0149800. doi: 10.1371/journal.pone.0149800 (PMC4768836; doi:10.1371/journal.pone.0149800)

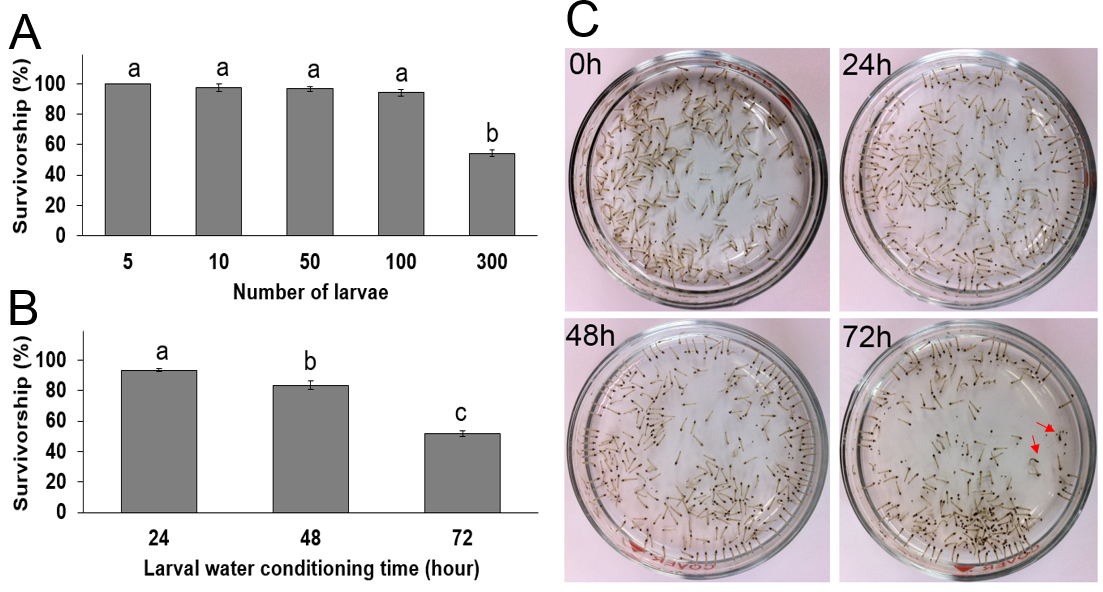

Supplement: S1 Fig — (A) Different number of larvae were starved for 72h and (B) 300 larvae were starved for differing time period. Differing letters indicate statistical difference at p = 0.05 (ANOVA, Tukey post-hoc HSD test). (C) Visual observation of 300 larvae held in 20 ml HPLC water without larval food at four different time points. Red arrows indicate examples of dead larvae at 72h time point. (TIF) [file pone.0149800.s001.tif]

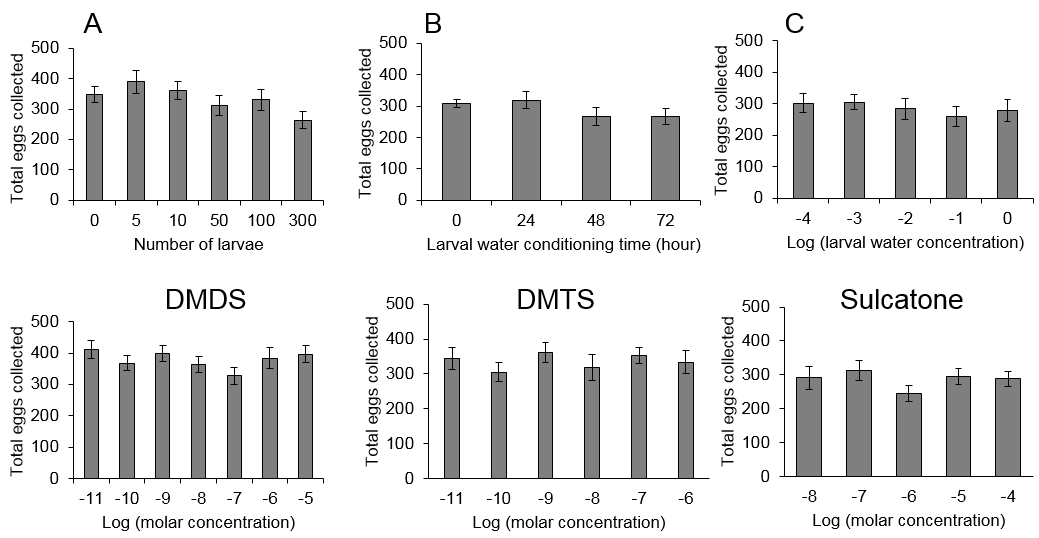

Supplement: S2 Fig — Number of total eggs did not differ by treatment variables (e.g., larval water treatments, concentration of test compounds, etc.). Error bar = s.e.m. Refer number of replicates from Fig 1 and Fig 3. (TIF) [file pone.0149800.s002.tif]

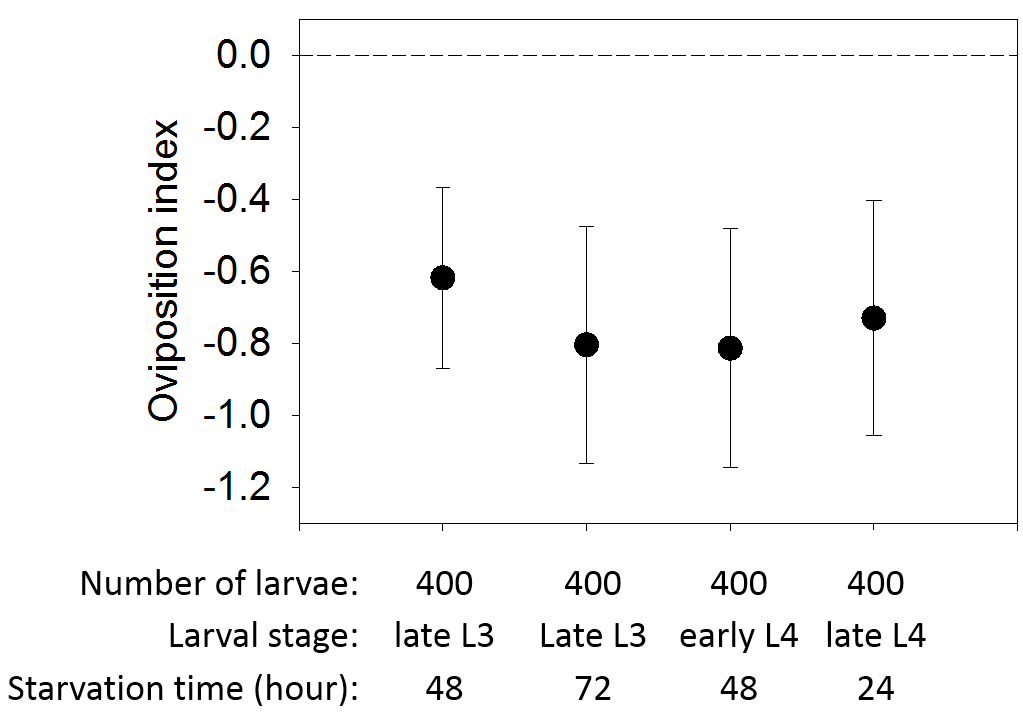

Supplement: S3 Fig — Additional bioassays using varied treatments in number of larvae, age of larvae, and conditioning time showed similar degree of repellent effects as shown in Fig 1. Error bar = s.e.m. (n = 5 ~ 6). (TIF) [file pone.0149800.s003.tif]

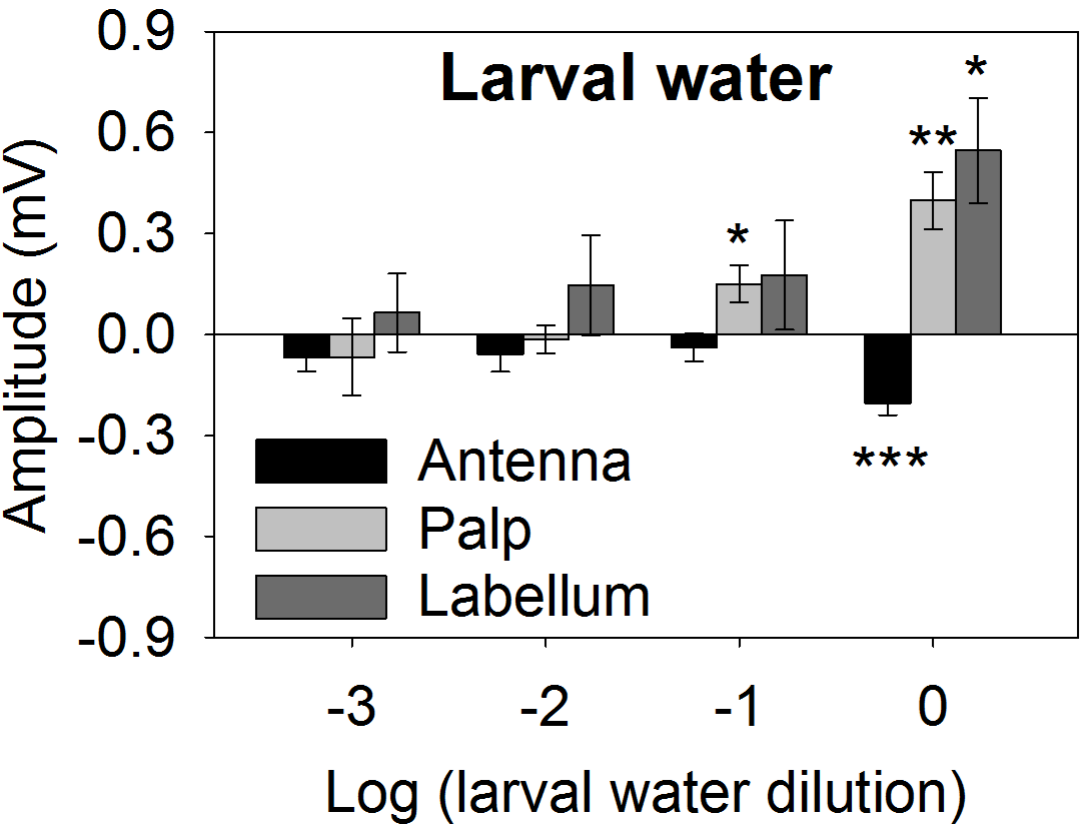

Supplement: S4 Fig — Responses are expressed as response difference to water control (ddH2O) of An. coluzzii females to larval water (300 larvae incubated for 72 h). Y axis represents response amplitude subtracted by control values and X axis represents log transformed larval water dilution. Asterisks represent significant response amplitude different from zero (***, p < 0.001; **, p < 0.01; *, p < 0.05; one sample t-test, one-sided). Error bar = s.e.m. (n = 7). (TIF) [file pone.0149800.s004.tif]

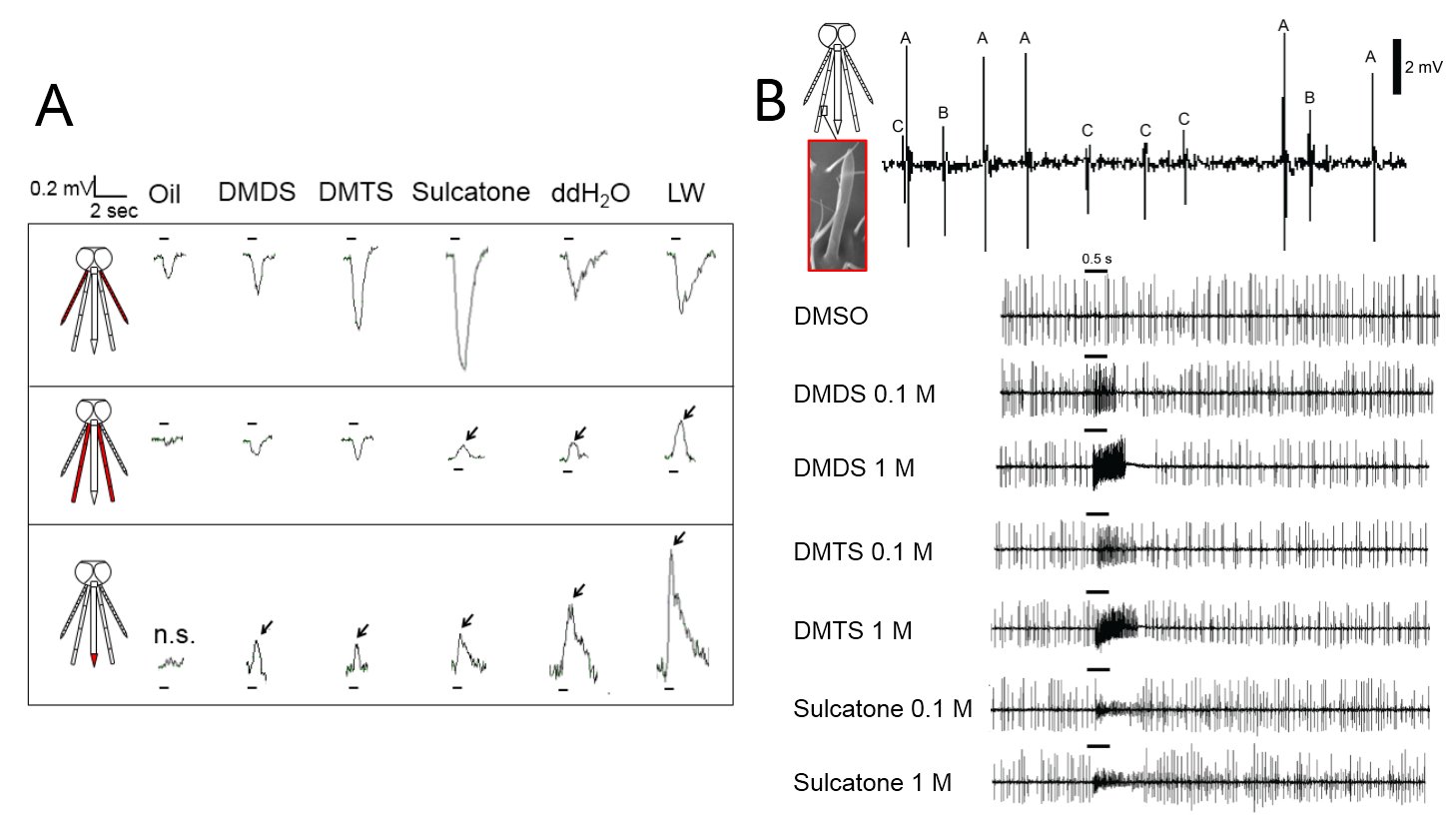

Supplement: S5 Fig — (A) Differential response kinetics for each odorant (10−1 M or undiluted standard larval water)–chemosensory organ combination in EAG/EPG/ELG (top to bottom; each chemosensory organ is highlighted in red in a schematic diagram of mosquito head) and arrows indicate upward responses. (B) Single-sensillum recordings of the responses of the maxillary palp capitate peg sensilla (highlighted in a red box; picture modified from [35]) of gravid An. coluzzii females to DMSO, DMDS, DMTS and sulcatone. Action potentials from different neurons are labelled A, B, or C according to spike amplitude and shape. Dark horizontal and vertical bars represent the 500 ms stimulus and 2 mV amplitude, respectively. (TIF) [file pone.0149800.s005.tif]
